# The effect of 12 weeks of combined training on hepatic fat content and metabolic flexibility of individuals with non-alcoholic fatty liver disease: Protocol of an open-label, single-center randomized control trial

**DOI:** 10.3389/fnut.2022.1065188

**Published:** 2023-01-16

**Authors:** Wei Huang, Weiqi Ruan, Cuilan Huo, Yanyu Lin, Tian Wang, Xiangdi Dai, Haonan Zhai, Jiasheng Ma, Jingyi Zhang, Jin Lu, Jie Zhuang

**Affiliations:** ^1^Shanghai Frontiers Science Research Base of Exercise and Metabolic Health, Shanghai University of Sport, Shanghai, China; ^2^School of Exercise and Health, Shanghai University of Sport, Shanghai, China; ^3^Department of Endocrinology, The First Affiliated Hospital of the Naval Medical University, Shanghai, China; ^4^School of Physical Education, Shanghai University of Sport, Shanghai, China; ^5^School of Elite Sport, Shanghai University of Sport, Shanghai, China

**Keywords:** NAFLD, metabolic flexibility, exercise intervention, combined training, randomized controlled trial, hepatic fat content

## Abstract

**Introduction:**

Metabolic flexibility (MetF) is the capacity of an organism to oxidate substrate according to substrate availability or demand. The mismatch of substrate availability and oxidation may cause ectopic fat accumulation in the muscle and the liver. The objectives of the study are to examine the effect of 12 weeks of combined exercise on hepatic fat reduction and investigate metabolites related to MetF before and after the high-fat diet between individuals with NAFLD and healthy control with an active lifestyle.

**Methods:**

This study is an open-label, single-center trial randomized controlled clinical study plus a cross-sectional comparison between individuals with NAFLD and healthy control. Individuals with NAFLD were allocated into two groups receiving resistance training (RT) combined with high-intensity interval training (HIIT) or moderate-intensity continuous training (MICT). Anthropometric indicators, clinical blood markers about glucose, lipid metabolism, and hepatic fat content (HFC) were assessed before and after the intervention. The metabolomics was also used to investigate the discrepant metabolites and mechanisms related to MetF.

**Discussion:**

Metabolic flexibility reflects the capacity of an organism to switch the oxidation substrates flexibly, which is associated with ectopic fat accumulation. Our study aimed to explore the discrepant metabolites related to MetF before and after a high-fat diet between individuals with NAFLD and healthy control. In addition, the study also examined the effectiveness of RT combined with HIIT or MICT on hepatic fat reduction and quantificationally analyzed the metabolites related to MetF before and after the intervention. Our results provided a perspective on fatty liver-associated metabolic inactivity.

**Trial registration:**

ClinicalTrials.gov: ChiCTR2200055110; Registered 31 December 2021, http://www.chictr.org.cn/index.aspx.

## 1. Introduction

The capacity of an organism to oxidate substrates according to the substrate availability or demand was described as metabolic flexibility (MetF) (1, 2). Metabolic inflexibility occurs in individuals with obesity or other metabolic diseases, which is manifested by a lowering of respiratory quotient (ΔRQ) before and after the euglycemic-hyperinsulinemic clamp (EHC) (1). MetF has been considered an indicator of metabolic health (3) since a progressive loss of MetF may be the cause of obesity-related comorbidities (4, 5).

Non-alcoholic fatty liver disease (NAFLD) is characterized by excess fat accumulation in the liver without excess alcohol intake (6). It covered approximately 25% of people worldwide (7) and 29.2% of people in China (8). Recently, NAFLD was termed as a metabolic (dysfunction)-associated fatty liver disease to reflect the disease feature (9, 10). Metabolic inflexibility is characterized by the mismatch of substrate oxidation and availability, which may be the etiology of insulin resistance (1). Thus, individuals with impaired MetF would form a set to favor the fat accumulating in the liver when they have a chronic high-fat diet or the increasing lipolysis of insulin-resistant adipose tissue (11, 12). However, there is little evidence about the causality between MetF and NAFLD. The cross-sectional study demonstrated that individuals with NAFLD show a typical feature of impaired MetF. Croci et al. reported that adults with NAFLD have lower ΔRQ (fasting and after stimulation of EHC) compared to healthy controls (13), and a similar result was also found in adolescents with NAFLD (14). In addition, even among obese individuals, those with NAFLD showed lower MetF than those without (15), and the hepatic and whole-body fat oxidation is reduced with the increase in hepatic fat content (13). Gastaldelli considered that the reduction of MetF in individuals with NALFD is a protective mechanism against excess FFA (16), and hyperglycemia will occur when the compensation mechanism is defective for the substrate overflow (16, 17). Thus, the response to substrate flux may play an important role in the development of NAFLD. Rudwill et al. found that metabolic inflexibility to a high-fat diet was preceded by whole-body glucose intolerance (18). Beyage et al. compared the different stimulations for MetF assessment and found that metabolic inflexibility to a high-fat diet is the only significant predictor for weight gain (5). In addition, Galgani et al. suggested that a high-fat diet is an advisable stimulating method for assessing MetF to lipid (19). Similarly, Fritzen et al. considered that the impaired response to FFA flux is a characteristic of obesity, and methods for improved FFA oxidation may be helpful for weight loss (20). However, little is known about MetF to a high-fat diet in individuals with NALFD, and there are few reports about the different metabolites related to MetF to a high-fat diet between individuals with NAFLD and in people with a healthy lifestyle.

Exercise intervention is the first-line method for treating metabolic disease, which is also an effective method for improving MetF (21, 22). The beneficial effects of exercise on hepatic fat reduction may depend substantially on the energy deficit (23), and a meta-analysis has demonstrated that high-volume exercise was superior to low-volume high-intensity exercise (24). However, a recent meta-analysis concluded that the effect of high-intensity interval training (HIIT) and moderate-intensity continuous training (MICT) on hepatic fat reduction was comparable (25). Furthermore, Hashida et al. found that resistance training (RT) improves NAFLD with less energy (26). Therefore, in addition to the energy deficit caused by exercise, some other pathways are mediating the association between exercise intervention and hepatic fat reduction. Both aerobic and resistance training are beneficial for hepatic fat reduction, and they all have their unique health benefits, such as improving cardiorespiratory fitness or muscle fitness. Combined training may be the ideal modality to gain the maximum health benefit, and Morze and colleagues reported that combined training may be the best modality for improving obesity (27), but little evidence about the effect of this exercise modality on hepatic fat content is available (28). The current physical activity guideline recommended that individuals with metabolic disease should perform both RT and moderate or vigorous physical activity (29), which could be implemented effectively by combined exercise. However, little is known about whether RT combined with HIIT would generate a similar effect on hepatic fat reduction.

Based on the aforementioned background about the MetF and NAFLD, some issues need to be explored. Thus, the objectives of our study are to examine the discrepant metabolites related to MetF to a high-fat diet in individuals with NAFLD and healthy control. In addition, we will compare the effect of different combined exercises on hepatic fat reduction and also analyze the metabolites related to MetF quantitively before and after the exercise intervention. Our results will provide evidence for clinical practice in choosing different combined exercises, and the results also provide a perspective on fatty liver-associated metabolic inflexibility.

## 2. Methods and analysis

### 2.1. Study design

**Figure 1:**
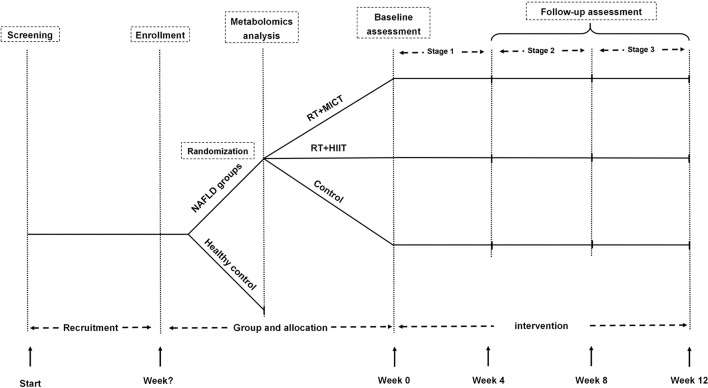
Flow diagram. RT, resistance training; HIIT, high-intensity interval training; MICT, moderate-intensity continuous training; NAFLD, non-alcoholic fatty liver disease.

This is an open-label, single-center trial randomized controlled clinical study. This study was approved by the ethics committee of Shanghai University of Sport (Number: 102772021RT08), and this trial has been registered in the Chinese Clinical Trial Registry (Number: ChiCTR2200055110, Registered 31 December 2021). This study recruited individuals with NAFLD and healthy individuals with active lifestyles. A cross-sectional comparison of MetF and related metabolites was conducted between individuals with NAFLD and the healthy control group. Subjects with NAFLD will be assigned into resistance training (RT) plus moderate-intensity continuous training (MICT) or high-intensity interval training (HIIT), and the control group in a 1:1:1 ratio. The subjects in the exercise group undergo a 12-week supervised exercise intervention based on their previous physical activity and diet habits, and the control group was asked for maintaining their previous lifestyles (Figure 1).

The assessment included two visits. First, participants who signed informed consent were invited for gathering the anthropometric information, and the exercise risk scan was performed by Pre-Activity Readiness Questionnaire plus (PARQ+) (30). Whereafter, the hepatic fat content (HFC) and cardiorespiratory fitness were assessed.

On the second visit, subjects were asked to arrive at the laboratory at 7:00–7:15 am. All subjects were advised to refrain from intensive physical activity, caffeine intake, and taking medicines for 24 h before each visit. The physical activity was investigated by the International Physical Activity Questionnaire short (IPAQ-short) (31, 32) and the 3-day diet before the test was also recorded by a photograph. Participants consumed a high-fat diet (consisting of 44% CHO, 41% fat, and 15% protein) within 15 min of collecting the fasting blood sample (18). Blood samples were collected at 30, 60, 90, 120, and 180 min. Respiratory quotient (RQ) was evaluated by an indirect calorimeter (ParvoMedics TrueOne 2,400 Metabolic Measurement System) before and 90 min after a high-fat diet. The substrate oxidation was calculated by the Frayn equation as follows (33):


$$\begin{array}{l} Fat\;oxidation\;\left(g/min\right)=1.67VO2\;\left(L/min\right)\\ -1.67VCO2\;\left(L/min\right)\\ Carbohydrate\;oxidation\;\left(g/min\right)=4.55VO2\;\left(L/min\right)\\ -3.21VCO2\left(L/min\right) \end{array}$$

### 2.2. Participants

Individuals who satisfied the following criteria were included in this study: (1) men or women aged 18–45 years who were overweight, obese (24 ≤ BMI < 35), or had central obesity (waist circumference ≥ 90 cm for men and ≥ 85 cm for women); (2) those who were diagnosed with NAFLD by ultrasonography and had ongoing or recent alcohol consumption of < 30 g (~10 g of alcohol per one drink unit) for men and < 20 g for women on average per week (7); (3) those who had no chronic cardiovascular disease or other diseases that prevent participation in exercise (assessed by doctors); (4) those who had stable drug consumption for the past 3 months (kind and dose); and (5) those who took no regular exercise in the past year (< 3 times/week and 30 min/time). The individuals were excluded if they (1) were being treated using insulin; (2) had unstable body weight (change ≥5 kg); (3) had uncontrollable blood pressure or glucose levels, or rapidly progressing disease; and (4) were unable to exercise due to any reasons.

### 2.3. Randomization and allocation

Eligible participants were assigned in a 1:1:1 ratio to undergo a 12-week intervention in the RT+MICT group (*n* = 18), RT+HIIT (*n* = 18) group, and the control group (*n* = 18), respectively. Computer-based randomization (www.radomization.com) was used with a block randomization design, and the block was defined by age (18–25, 26–30, 31–35, 36–40, or 41–45) and sex (male or female). Before the intervention, the allocation was concealed in opaque envelopes and drawn by the participants.

### 2.4. Intervention

After all the assessments, participants in the two intervention groups spent a week acclimating, which consisted of teaching the correct movements, assessing the one-repetition maximum (1RM) of each movement, and acclimatizing at 50–60% 1RM. For safety reasons, the 1-RM load was estimated at 10 RM according to the conversion table (34), and the contracting and relaxing course lasted 3–4 s to recruit more muscle fiber and prevent injury. The control group received usual care without additional exercise guides and intervention, and they were promised to obtain the same intervention after 12 weeks to increase compliance. Two intervention groups performed a total of 36 sessions of RT+MICT or RT+HIIT in the next 12 weeks, and each session consisted of 5 min of warm-up and stretching; (2) 30 min of resistance training and 30 min of MICT or 15 min of HIIT; and (3) 5 min of cool down. RT was performed on fixed strength training machine (Life Fitness, Illinois, US), consisting of 1–2 sets with 10–15RM for 10 movements, including chest press, shoulder press, seated pull-down, row, leg curl, leg press, leg extension, glute, abdominal curl, and prone raise. The MICT and HIIT were performed on a treadmill, elliptical machine, cycle ergometer, or rowing machine. MICT was performed at 40–60% heart rate reserve (HRR) for 30 min, and the HIIT protocol consisted of 3× 3 min of high-intensity intervals at 70–90% HRR, interspersed with 2 min of active recovery. Table 1 visualizes the intervention protocol. The exercise intensity of MICT and HIIT was monitored by Polar OH1 with a pad, which could display the heart rate in real-time.

**Table 1:** Details of the exercise protocol.

**Stage**	**Exercise protocol**
Stage 1	**RT**: 1 Set, 15 RM, 10 Movements **MICT**: 45–55% HRR for 30 min with treadmill, ergometer or elliptical **HIIT**: 40% HRR * 2 min**/** 70% HRR * 3 min
Stage 2	**RT**: 1 Set, 10 RM, 10 Movements **MICT**: 55–60% HRR for 30 min with treadmill, ergometer or elliptical **HIIT**: 40% HRR * 2 min **/** 80% HRR * 3 min
Stage 3	**RT**: 2 Sets, 10 RM, 10 Movements **MICT**: 55–60% HRR for 30 min with treadmill, ergometer or elliptical **HIIT**: 40% HRR * 2 min**/** 90% HRR * 3 min

The exercise intervention is divided into three stages, and each stage contains 4 weeks. RT, resistance training; MICT, moderate-intensity continuous training; HIIT, high-intensity interval training.

All instructors received uniform training before the intervention, including the whole intervention process (Table 1), movement standard (e.g., when doing leg extension, the calf straight is counted), and how to fill in the training record.

### 2.5. Outcome assessment

#### 2.5.1. Primary outcome

The primary outcome of this study is the hepatic fat content, which is assessed by Siemens 3T Magnetom Prisma scanner (Siemens Medical Solutions, Erlangen, Germany).

#### 2.5.2. Secondary outcomes

Secondary outcomes include anthropometric indicators (body weight, body mass index, and the circumference of the waist and the hip), body composition (body fat, body fat percentage, lean body mass, abdominal subcutaneous, and visceral fat), physical fitness (grip strength and peak oxygen uptake), clinical blood markers (glucose, insulin, non-esterified fatty acid, blood lipid profile, and liver enzyme), metabolic flexibility (the change of respiratory quotient before and after the high-fat diet), and target and untargeted metabolomics, which explore the discrepant metabolites related to MetF between individuals with NAFLD and healthy control and quantitively analyze the change of these metabolites before and after the exercise intervention. In addition, physical activity, dietary habits, and sleep were investigated by questionnaires. The details of the assessing method are in the Supplementary material. The schedule of study assessment is presented in the Table 2.

**Table 2:** Schedule of study assessment.

**Variables**	**Screening visit**	**Baseline assessment**	**Week 4**	**Week 8**	**Week 12**
Informed consent	•				
Drugs investigation	•	•	•	•	•
Physical activity	•	•			•
Dietary investigation		•	•	•	•
Anthropometrics	•	•	•	•	•
Body composition		•	•	•	•
Physical fitness		•	•	•	•
Hepatic fat content		•			•
Abdominal visceral and subcutaneous fat		•			•
Clinical blood markers		•			•
Metabolic flexibility		•			•
Metabolomics		•			•

### 2.6. Concomitant adherences and procedures

To increase adherence, we organized the participants into collaborative groups according to the time of participation, and one instructor was assigned to each training group. The instructors notified them *via* WeChat before the agreed training time. In addition, we provided training feedback regularly and gave a reward according to the phased attendance each month. The attendance time was recorded in a spreadsheet, and the adherence was calculated as attendance time divided by total training times. The time points and reasons for dropping out and withdrawing were also recorded.

### 2.7. Sample size estimation

The sample size calculation was based on the results of a previous study (35), and we calculated the effect size as 0.8 based on accessible data. The effect size was estimated conservatively, with an effect size of 0.4 in our study. At least 42 participants were needed to provide 95% statistical power with a two-tail 0.05 significance level when using the one-way ANOVA and to keep the ratio 1:1:1 in the three groups. With an attrition rate of 20%, a total of 54 participants need to be recruited (G^*^power 3.1). The calculating process in G^*^power is in the Supplementary material. At least 10 healthy active individuals were recruited for metabolomic analysis, and this sample size was enough for metabolomic analysis (36).

### 2.8. Statistical analysis

The continuous variables (ΔRQ, blood pressure, glucose, etc.) were presented as means and standard deviations, and discrete variables were presented as numbers and percentages (e.g., sex). The Shapiro–Wilk test was used to test the normality of the continuous variables. For the continuous data to fit the normal distribution, the Student's *t*-test was used to compare the difference between the two samples (two groups in baseline, or before and after intervention), or the Wilcoxon rank sum test was used, and the Chi-squared test was used for categorical data. The covariance analysis was used to examine the difference between clinical and metabolomics data, with the baseline level as the covariate. In addition, the intention-to-treat analysis was conducted by a linear mixed-effect model (LMM), which considers the missing data as randomized and does not impute the missing data (37). The sensitivity analysis is performed to verify the robustness of the results. Age, sex, and diabetic or not were included in the LMM when comparing the main clinical indicators, and we included the baseline variables that differed between the dropout participants and those who completed the study as additional covariates in the LMM of the primary analysis. This method helped in decreasing the bias and provided a more realistic data analysis. A two-tailed test was conducted, and a *p* < 0.05 was regarded as a statistically significant difference. The data analysis was performed by the JMP pro version 16 (SAS Institute Inc., Cary, NC). For metabolomics analysis, the raw data were transformed to centroid mode and mass-corrected before being analyzed using the XCMS platform. The preprocessed data were imported into excel for normalization, and a two-dimensional matrix was formed. A principal component analysis and an orthogonal partial least squares discriminant analysis (OPLS-DA) were performed using the SIMCA 13.0 software (Umetrics AB, Malmo, Sweden). The identification of discrepant metabolites was according to the standard by variable weight (VIP)>1 and *p* < 0.05 in the OPLS-DA model. The correlation between discrepant metabolites and MetF was analyzed using Spearman's correlation and partial correlation analysis, and the change of the discrepant metabolites before and after interventions was analyzed by the Student's *t*-test.

## 3. Discussion

NAFLD is a chronic liver disease that affects approximately 25% of the population worldwide (7), and metabolic inflexibility may be the factor for the occurrence of NAFLD (8). Our study aimed to analyze the discrepant metabolites related to MetF when receiving a high-fat diet between individuals with NAFLD and healthy control with cardiorespiratory fitness, which has been found associated with MetF. In addition, we also compared the effect of different combinations of RT plus HIIT or MICT on hepatic fat reduction and examined the association between the changes of hepatic fat and the discrepant metabolites before and after the 12-week exercise intervention. In addition to examining the effect of different exercise combination on hepatic fat reduction, our study also provided an alternative perspective for NAFLD treatment, which improved the substrate metabolism rather than making an energy deficit.

### 3.1. Exercise, metabolic flexibility, and non-alcoholic fatty liver disease

Until now, there are no specific drugs for treating NAFLD, but exercise is the first-line method for treating this disease and preventing the progress of non-alcoholic fatty liver (22). On the one hand, exercise decreases the HFC by increasing energy consumption. The meta-analysis demonstrated that the effectiveness of exercise on hepatic fat reduction is mediated by weight loss (23). On the other hand, exercise decreases the HFC and may not be mediated by energy deficit completely because Hallsworth et al. have found that resistance training decreases the HFC without weight loss (38). An impaired MetF in response to a high-fat diet will cause more weight gain in future (5), and the high-fat diet will cause more hepatic fat accumulation and metabolic inflexibility (39). In addition, physical inactivity is one of the impaired capacities in response to high-fat diet stimulation of healthy individuals, followed by a decrease in insulin sensitivity (18). Therefore, the metabolic inflexibility to a high-fat diet caused by physical inactivity may be one of the etiologies of NAFLD. Exercise intervention is an effective method for increasing metabolic flexibility, whose core is the mitochondrial function (40). Thus, exercise intervention decreased hepatic fat reduction, which may be mediated by metabolic flexibility improvement partially.

Combined exercise also termed concurrent exercise is an effective method for meeting the physical activity guideline (41). Although different exercise modalities have specific adaptions, such as resistance exercise increasing muscle strength and mass and aerobic exercise increasing the capacity to intake and utilize oxygen, both resistance exercise and aerobic exercise are effective for improving metabolic health. Bacchi et al. reported that 40 patients with T2DM were randomly assigned to aerobic training or resistance training groups; aerobic training improved the peak oxygen consumption and resistance training increased the muscle strength, but they demonstrated a similar effect on HbA1c decreasing (42). Furthermore, Hashida and colleagues systematically reviewed the effectiveness of these two-exercise modalities on hepatic fat reduction and reported that resistance training decreases hepatic fat similar to aerobic training with less energy consumption (26). However, there may be some interference effect in training adaption when performing resistance training and aerobic training concurrently (41, 43), due to the distinct training adaption, while it may be more effective in improving the metabolic health of individuals with metabolic dysfunction (27, 44) and untrained individuals (45, 46). HIIT is an exercise modality characterized by repeated bouts of high-intensity effort interspersed with recovery periods (47). It has been demonstrated that HIIT could decrease abdominal fat (48) and cause similar effects on body composition (49) and hepatic fat content with MICT (25) but with less time and energy consumption. As described in the introduction, exercise decreases hepatic fat may be independent of energy consumption partially. Although many investigations have examined the effectiveness of a single exercise modality on hepatic fat reduction, few studies have compared the different combinations of resistance training plus moderate-intensity continuous training or high-intensity interval training (28, 50, 51). In the current physical activity guidelines, individuals with chronic diseases need to perform moderate-intensity aerobic physical activity or vigorous-intensity aerobic physical activity combined with muscle-strengthening activity. Thus, the study provided some evidence about the adaption of different exercise combinations with a randomized control design.

However, the biggest challenge of our study was to motivate the volunteers with a sedentary lifestyle formerly adhere to exercise. For this issue, we took some strategies for behavior change, such as regular notice before each exercise session, setting a personal goal, and assessing completion regularly. In addition, a regular evaluation was conducted and an award was presented for a higher attendance rate. On the contrary, supervised training was important. First, the subjects in our study were untrained people, so they did not know how to start exercising. Exercise, especially resistance exercise, had a high injury risk without correct movement, and they would not recruit the correct muscle without specialized support. Furthermore, the subjects in our study are at moderate-to-high exercise risk; hence, they need specialized personnel to supervise their training.

### 3.2. Metabolomics and non-alcoholic fatty liver disease

Metabolomics is a method to examine the small molecules and metabolic products, such as amino acids, fatty acids, and carbohydrates, which have been applied widely to explore the marker for diagnosis and pathophysiology (52). Dietary triglycerides cover 15% of the total fat accumulated in the liver in individuals with NAFLD (53). Thus, exploring the discrepant metabolites in fasting and after a high-fat diet stimulation among individuals with NALFD and healthy control with good cardiorespiratory fitness may help in understanding the pathophysiology of NAFLD. Individuals with good cardiorespiratory fitness are a good model for understanding the mitochondrial capacity on substrate metabolism since cardiorespiratory fitness is the reflection of mitochondrial capacity (54). Although elite endurance athletes are superior metabolic models, it is not realistic for our participants to reach this level. Thus, recruiting individuals with good CRF as the control is suitable. Yu et al. reported that the pathway of the tricarboxylic acid cycle, primary bile acid biosynthesis, and linoleic acid metabolism was significantly different at fasting and after the mixed-meal challenge, but they did not correlate these pathways to physiological function (MetF) (55). However, few studies have explored the association between HFC and postprandial metabolomes (56). In addition, whether there is some correlation between training effectiveness and postprandial metabolic features like gut microbiotas is still unclear (57). We also quantitively analyzed the change of discrepant metabolites before and after the exercise intervention, which helped us understand the effectiveness of exercise on substrate metabolism. In addition, it also contributed to the development of new drugs or treatment methods.

Summarily, if our study implements successfully, it not only provides evidence for combined training in hepatic fat reduction but also provides an alternative perspective for exercise to improve metabolic health.

### 3.3. Limitation

We did not use the oral glucose tolerance test (OGTT) or euglycemic-hyperinsulinemic clamp (EHC) as the stimulating method for assessing metabolic flexibility (MetF), which are the standard methods for measuring the insulin sensitivity and have the reference standard. However, there is no standard assessing method for MetF. In addition, the study from Galgani et al. and Fritzen et al. emphasized the importance of a high-fat diet in assessing MetF (19, 20). Thus, we chose the high-fat diet as the stimulating method for MetF assessment. Second, our study did not control the diet strictly, which limits the effectiveness of our intervention on weight loss. However, it is challenging to limit energy intake and kinds of diets. Our study adds an exercise intervention to daily life with regular diets and drug use and asks the subjects to maintain their everyday life. Thus, our study will provide evidence of the effectiveness of exercise intervention in real-world conditions. Third, our intervention of HIIT and MICT is not energy-matched. In daily life, individuals would not participate exercise with a precise energy monitor. In other words, the limitation for individuals conducting a training plan is not energy consumption but time. Thus, we design the HIIT protocol with a half-time of MICT, rather than half of the energy consumption of MICT.

## Ethics statement

The studies involving human participants were reviewed and approved by Shanghai University of Sport Ethics Committee. The patients/participants provided their written informed consent to participate in this study.

## Author contributions

WH, WR, JL, and JZhu: study design and manuscript drafting. CH: recruitment, sample collection, and writing. JL and JZhu: project administration, design, data collection, data analysis, writing, and supervision. XD, HZ, JM, and JZha: daily administration of volunteers and performing the training. YL and TW: data collection, data management, and data analysis. All authors revised the final version of the protocol manuscript, read, and approved the final manuscript.
