# Supplementary material for: The effect of 12 weeks of combined training on hepatic fat content and metabolic flexibility of individuals with non-alcoholic fatty liver disease: Protocol of an open-label, single-center randomized control trial
Source: Front Nutr. 2023 Jan 16;9:1065188. doi: 10.3389/fnut.2022.1065188 (PMC9884837; doi:10.3389/fnut.2022.1065188)
Supplement: Supplementary file 1 [file Table_1.DOCX]

Supplementary Material

1.1 Measurement and Evaluations

## Anthropometric assessment

Bodyweight and height will be measured by a stadiometer (TANITA, Japan). Waist circumference will be measured at the midpoint between the lower costal margin and the level of the anterior superior iliac crests. The hip circumference will be measured at the level of the greater trochanter using a tape measure. Body composition is measured by dual-energy X-ray absorption determination (Lunar prodigy GE health, US).

## Blood samples

Blood samples will be collected by vacuum tube without coagulation accelerator and anticoagulation from venous blood after overnight fasting, and 30min, 60min, 90min, 120min, and 180min after mixed meal stimulation. The whole blood will be set in indoor temperature(26℃) for 30 minutes, then centrifuged with 2500g for 15min at 4℃, and the partial blood samples are collected by heparin sodium-anticoagulant tubes for plasma sample. The supernatant serum will be extracted and divided into aliquots and stored at -80℃ until for analysis. Total cholesterol, high-density lipoprotein cholesterol (HDL-c), low-density lipoprotein cholesterol (LDL-c), triglycerides, apolipoproteins A-I and B, glucose, insulin, free fatty acid are be measured from serum sample by conventional methods. In addition, the plasma samples will be used in metabolomics by liquid chromatography-mass spectrometry (LC-MS), which will be used to explore the mechanism of difference in metabolic flexibility.

## Physical activity, sleep and dietary Assessment

Physical activity and diet habits are two important factors influence the effectiveness of exercise intervention and the assessment of metabolic flexibility, so these two factors need to be investigated as control variables. Physical activity will be assessed using IPAQ-short before baseline and follow-up assessments. The sleep quality of subjects will be assessed by Pittsburgh Sleep Quality Index(PSQI) questionnaire, a general and reliable method for sleep investigation and has already been localized(1). In addition, three days of diet before baseline and follow-up assessment are recorded by a commercially available mobile application (Bohe health APP, Shanghai Bohe health technology Co., Ltd, Shanghai, China). The app will be used to record all the food intake, including meals, snacks and beverages. Subjects input the kind and volume(reference by an credit card) of food, and take a photograph(Figure 1). The app will calculate the calories and nutrient composition according to the China Food Composition Tables(1). The APP has been used in previous weight management program for Chinese adults(2). Participants will be asked to consume similar food according to the photograph to avoid influencing the metabolic flexibility assessment. In addition, the sample food frequency questionnaire(FFQ 25) will be used to examined the change of diet habits(3). The FFQ25 include the 25 kinds of common foods, and assessing the quantity by referencing to hand, The reliability and validity has been examined in Shanghai previously(4).


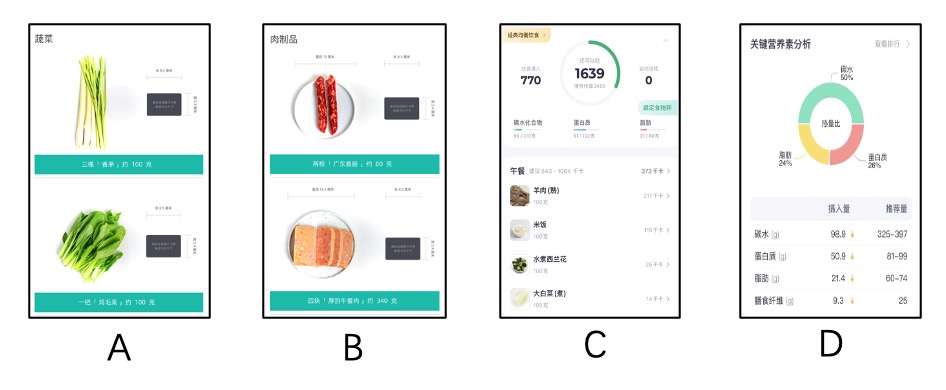


Figure 1. The operation interface of BOHE APP.

The weight estimation of the food, vegetables(A) and meat(B). The energy estimation of total intake(C), and the ratio of macronutrient(D).

## Muscle strength

A grip dynamometer (Takei T.K.K 5401, Japan) will be used to assess the grip strength, which partially reflects the whole-body strength. Participants will be asked to remain in the upright position with their arms lowered, then hold the grip dynamometer with a suitable width. Subsequently, they will be asked to try their best for the strength test and alternate their left and right hands twice. The average of the maximal values from both the hands will be recorded (5).

## Cardiorespiratory fitness

Cardiorespiratory fitness capacity will be assessed using graded exercise testing (GXT) on a cycle ergometer (Monark 825E, Varberg, Sweden) to evaluate the peak oxygen uptake (VO_2peak_) by Trueone 2400 under the supervision of a trained exercise physiologist. Following 5 minutes of stretching, participants start pedaling at 35 W for women and 50 W for men for 3 minutes. Subsequently, the load increase by 15 W every 60 seconds until subjective exhaustion occurs, and the data will be collected every 10 seconds. VO2peak will be determined by using the average of the highest value in 30 seconds. Heart rate will be monitored using an electrocardiogram at rest and during the test.

## Metabolic flexibility

Metabolic flexibility will be assessed by indirect calorimeter with a high-fat diet stimulation. The basal metabolism will be evaluated before the high-fat diet with a supine position lasting for 20 minutes, and the postprandial metabolism is evaluated during 90- to 120-minutes after the high-fat diet. The metabolism flexibility will be presented by change of respiratory quotient(RQ) before and after the high-fat diet stimulation(6). The first 5-minutes metabolic data will be removed for the stability of results.

## Metabolomics

In addition to the target metabolomics, untargeted metabolomics based on liquid chromatography-mass spectrometry(LC-MS) will be used to analyze plasma metabolites associated with MetF using the plasma 2 hours after the high-fat diet. We will also compare the MetF related metabolites between individuals with NAFLD and healthy control. In addition, we will also compare the MetF related metabolites before and after the exercise intervention, and examine the association between these metabolites and hepatic fat reduction. The raw data will be transformed to centroid mode and mass corrected before being analyzed using the XCMS platform. The preprocessed data will be imported into Excel for normalization, and a two-dimensional matrix will be formed. Principal component analysis and orthogonal partial least squares discriminant analysis (OPLS-DA) will be performed using the SIMCA 13.0 software. The identification of different metabolites will be according to the standard by variable weight (VIP)>1 and p-value < 0.05 in the OPLS-DA model.

## Hepatic fat content and abdominal fat assessment

Magnetic resonance imaging will be used to assess the abdominal fat and hepatic fat content(HFC), which will be performed using a Siemens 3T Magnetom Prisma scanner (Siemens Medical Solutions, Erlangen, Germany). A combined sequence Siemens Liverlab will be used to assess the abdominal fat and HFC. For abdominal fat assessment, axial T1-weighted dual-echo (in-phase and out-of-phase) volumetric interpolated breath-hold examination (VIBE) images are obtained for the entire abdominal area while holding deep expiration and applying the following parameters: repetition time (TR) 3.97 ms, first echo time (TE1) 1.29 ms (out-of-phase) and second echo time (TE2) 2.52 ms (in-phase), slice thickness 3.0 mm, voxel size 1.2×1.2×3.0 mm, distance factor 20%, matrix 320×208, field of view (FoV) 45 cm, flip angle (FA) 9 degrees, and average acquisition time of 15 seconds. The OsiriX software package (OsiriX Foundation, Geneva, Switzerland) is used to segment the abdominal subcutaneous, visceral, and retroperitoneal fat. In addition, the VIBE Multi-echo Dixon sequence will be used to assess the HFC using the following parameters: TR 9.0 ms, TE1 1.09 ms, numbers of averages 1.0, slice thickness 3.5 mm, voxel size 1.2×1.2×3.5 mm, FoV 45 cm.

Sample size calculating process:

**F tests** - ANOVA: Repeated measures, within-between interaction

**Analysis:** A priori: Compute required sample size

**Input:** Effect size f = 0.4

α err prob = 0.05

Power (1-β err prob) = 0.95

Number of groups = 3

Number of measurements = 2

Corr among rep measures = 0.2

Nonsphericity correction ε = 1

**Output:** Noncentrality parameter λ = 16.8000000

Critical F = 3.2380961

Numerator df = 2.0000000

Denominator df = 39.0000000

Total sample size = 42

Actual power = 0.9511180

**Reference**

Buysse, D. J., Reynolds, C. F., Monk, T. H., Berman, S. R., & Kupfer, D. J. The Pittsburgh sleep quality index: A new instrument for psychiatric practice and research. Psychiatry Research(1989) , 28(2), 193–213. doi: 10.1016/0165-1781(89)90047-4

# Food Composition Database. https://nlc.chinanutri.cn/fq/ [Accessed December 20, 2022]

Yang X, Chattopadhyay K, Hubbard R, Li J-L, Li L, Lin Y. 36-Month Evaluation of a Weight Management Programme in Chinese Overweight and Obese Adults. *Front Public Health* (2021) 9:749302. doi: 10.3389/fpubh.2021.749302

Gao J, Fei JQ, Jiang LJ, Yao WQ, Lin B, Guo HW. Assessment of the reproducibility and validity of a simple food-frequency questionnaire used in dietary patterns studies. Acta Nutr. Sin. 2011;33:452-6. doi: 10.13325/j.cnki.acta.nutr.sin.2011.05.012

5. Hu S, Gu Y, Lu Z, Zhang Q, Liu L, Meng G, Yao Z, Wu H, Bao X, Chi VTQ, et al. Relationship Between Grip Strength and Prediabetes in a Large-Scale Adult Population. *Am J Prev Med* (2019) 56:844–851. doi: 10.1016/j.amepre.2019.01.013

6. Rudwill F, O’Gorman D, Lefai E, Chery I, Zahariev A, Normand S, Pagano AF, Chopard A, Damiot A, Laurens C, et al. Metabolic Inflexibility Is an Early Marker of Bed-Rest-Induced Glucose Intolerance Even When Fat Mass Is Stable. *J Clin Endocrinol Metab* (2018) 103:1910–1920. doi: 2020070111335603300
